# Collectanea Medica: Consisting of Anecdotes, Facts, Extracts, Illustrations, &c.

**Published:** 1826-05

**Authors:** 


### COLLECTANEA MEDICA:

CONSISTING OF

### ANECDOTES, FACTS, EXTRACTS, ILLUSTRATIONS, &c.

*Relating to the History or the Art of Medicine, and the Collateral Sciences.*

---

Floriferus, ut apes, in saltibus omnia libant,
Omnia nos, itidem, depascimur aurea dicta.

---

ART. I.—*Report made by* M. MAGENDIE *to the Academy of Sciences (at their Meeting, July* 1825,*) on the subject of a Boy, named Honoré Trezel, Deaf and Dumb from his Birth, who obtained Speech and Hearing under the care of* Dr. DELEAU, Jun. (From the *Edinburgh Journal of Medical Science*, No. ii.)

AT the sitting of the 10th May, 1824, M. Percy made known to the Academy, that a deaf and dumb boy, named Trezel, had lately acquired hearing under the care of M. Deleau. The success had been as complete as could be desired. The child, who before the operation was entirely deaf, had been enabled to hear all descriptions of noise, and even to notice certain intonations of the voice.

But Trezel was, notwithstanding, far from having acquired the knowledge of sounds, though he enjoyed the faculty of hearing. An immense interval still separated him from children of his own age, who were possessed of a good organisation. Noises of all kinds, the accent of the voice, the words which were addressed to him, as well as those which he endeavoured to form in his larynx, were only to him a source of new sensations which delighted him; but he drew no other use from them. He was ignorant of the great advantages of language, and perfectly unaware that the vague and strange sounds that he sometimes produced with his vocal organ, might be the means one day of enabling him to express his wants and his thoughts. A melancholy experience has, in other instances, shown that if such a deaf and dumb patient, under these circumstances, be merely left to the care of his family, his sense and his intelligence will remain in a state which is not much superior to that in which he found himself before his cure had been completed. As soon, therefore, as Honoré Trezel had obtained the faculty of hearing, such an education was required as might be a substitute for that which his infirmity had prevented him from receiving, and which should enable him to avail himself of the sense that he had just recovered so happily. At the meeting in which M. Percy announced the result of the operation performed upon the young Trezel, he added, that M. Deleau was engaged in the instruction of this child, and that he would make known the result of it to the Academy. M. Deleau has kept his promise.—Trezel was presented to you in one of your last sittings. He repeated from memory the fable of the Fox and the Crow, performed different exercises of analysis; and you have been enabled to judge yourselves of the state of his hearing, of his voice, and of his degree of intelligence, after nine months of assiduous care. This fact is so much the more important, as none of the deaf and dumb, who have obtained the faculty of hearing by an operation, or who have acquired it spontaneously, have been observed a sufficient length of time by men of information, who could inform us what change had been effected in them, by a new sense intervening all at once in the midst of senses already tried; or who could make known what alterations have taken place in the intelligence, the instinct, the speech, or the motions of such individuals, by the development of a function so important as that of hearing; or, lastly, who could acquaint us whether a person born deaf and dumb, and who has acquired hearing, is capable of entering into all the relations of social life, or whether he is not apt to step out of their bounds. It is evident that many interesting physiological questions connect themselves with the treatment of M. Deleau; and for this reason commissioners were appointed by you, who have considered it their duty to collect and verify all the circumstances of the case. What I am going to relate is an abstract of the report made on the occasion.

Claude Honoré Trezel, now ten years of age, born at Paris, of poor parents, was of that class of the deaf and dumb who did not even hear the most violent noises, or the loudest explosions. His forehead was large, and his head well made; but his physiognomy, the image of his intelligence, had little expression. He dragged his feet in walking; his gait was tottering; he did not know how to blow his nose; and had, in other respects, received no education adapted to his situation. He made his principal wants known by a definite number of signs.

The operation which was performed was not a new one; it was invented towards the end of the last century by a deaf person of Versailles, who, fatigued with his situation, succeeded in curing himself. It is now adopted by all physicians who treat disorders of the ear; it has, above all, been frequently employed in practice by Dr. Itard. It consists in injections of air, or of different liquids, into the drum of the tympanum, by the tube which ends in the back part of the mouth. It has many serious inconveniences, which happily did not present themselves in the case of young Trezel.*

The days which immediately followed an accession of hearing, were for Honoré a period of rapture. The noises which he heard gave him ineffable pleasure; he sought them with avidity: on hearing a musical snuff-box, he was in a sort of extasy. But there was a certain time required before he could perceive that speech was a medium of communication; besides he attended at first, not merely to the sounds which were formed, but to the movements of the lips by which they were accompanied; and thus he believed, during some days, that a child seven

* It has been explained, in a separate work published on this case, that injections of water, by means of a small flexible sound, were forced into both the Eustachian tubes. These injections were not either accompanied by those dreadful pains which sometimes determine fainting, and oblige the treatment to be suspended; or followed by abscess and suppuration in the drum, which resist all hope of cure.

months old spoke like grown-up persons, merely because he saw his lips make movements. But he was soon taught his error; and he was aware from that time that it was to the sounds he should attach importance, and not to the motions of the lips.

But it unfortunately happened that he heard a magpie pronounce some phrases. In generalising on this particular fact, he concluded that all animals were gifted with speech, and consequently was anxious to oblige a dog to speak, of which he was very fond. He even resorted to violence to make him say *papa*, *du pain*, the only words that he could himself yet pronounce; but the cries of the poor animal terrified him, and he desisted from his attempt.

These first periods of hearing produced a great change in the physical state of Trezel. His gait became more firm; the mournful expression of his face was changed into a smiling and gay air; he learned to blow his nose, and ceased to drag his feet in walking.

A month had passed away, and Honoré remained nearly at the same point. Absorbed by his sensations and his new remarks, he could not comprehend the different syllables which formed words; and nearly three months were necessary before he could distinguish some compound words, before he could know their sense, and that of short and simple syllables.

It was likewise a length of time before he knew the direction of sound. A person having concealed himself in the room where the boy was employed, called out to him, but it was with considerable difficulty Trezel discovered the hiding-place of the individual who hailed him; and this discovery was much more owing to the eye, and to the reasoning at which he had arrived, than to the employment of the ear.

All the interest, however, which Honoré felt in the sensations procured him by his hearing, had not prevented him from making one observation of the greatest importance,—his larynx formed sounds; and to the pleasure of hearing them was superadded that of being able to produce them. It was in this that Trezel's case displayed the most curious and the newest pheuomena.

The instrument of the voice is composed of a great number of different parts, among which are found muscles, bones, cartilages, and membranes; consequently, it would have been wonderful if, without some preparatory exercise, all these parts, all these organs, had acted at once in concert, so as to produce vocal sounds and appreciable articulation: this, as we ought to expect, did not happen. The first sounds that Trezel was enabled to utter were dull and heavy; he pronounced, not without difficulty, A, O, U. The two other vowels came much later; and the first words that he formed were *papa*, *tabac*, *du feu*, &c. But, when he wished to repeat more complicated words, he made a multitude of contortions of the lips, of the tongue, and of all the agents of pronunciation, of the use of which he was entirely ignorant, resembling, in this respect, a person who begins to learn the art of dancing or swimming, and who exhausts himself in useless efforts and ungraceful movements.

But, induced by temptations, he acquired the pronunciation of some compound words, which had at first been beyond his abilities.

It was at the moment of making this attainment, that he conceived himself on a level with other children of his own age. Satisfied with himself, therefore, and proud of his new situation, he treated his old companions in misfortune with great disdain, and wished to see them no more. Few persons who had seen him at this time would have discovered in him a happy disposition.

But, notwithstanding this little emotion of vanity, Trezel advanced slowly in pronunciation. He left out a great number of syllables, or rather he articulated them in a very defective manner. Perhaps he would never have overcome this difficulty, if recourse had been merely had to his organs of hearing, but an appeal was likewise made to his sight. Different syllables were delineated for him, and from this moment he pronounced them much better, comprehending with much more clearness the assemblage of vowels and consonants, and the reciprocal influence which these exert upon each other. Thus a very remarkable fact was proved,—namely, that the association of the sight with the movements of the larynx was prompt and easy, while that of the hearing and of the organ of the voice was always difficult, and was only exercised with slowness. For example, as soon as Honoré perceived written syllables, he pronounced them, if at the same time they were reverberated close to him; but, if the table on which the letters were traced was carried away, in vain were certain syllables articulated in his ear in the most distinct manner possible; to articulate them himself was an impossibility. He comprehended much more easily the affinity of sounds to written letters, than to the action of his larynx.

By constantly adhering to this mode of proceeding, Trezel has learned to read and write with sufficient rapidity, but after a manner which is similar to that of persons who learn a strange language, and who, in general, read it and write it long before they can speak it. Up to the present moment, Honoré reads with his eyes, and writes infinitely better than he speaks.

His pronunciation is very defective. The Rs in particular rumble in his mouth in a most singular and disagreeable manner. The different varieties of accent seem unknown to him; but, when we consider the point from which he set out, we ought to be fully satisfied to see in him the degree of instruction which he displays in so short a time.

Honoré exhibits another phenomenon, which has excited the attention of the commissioners of the Academy. When a word is said distinctly to him, he repeats it immediately; when he is hailed, for instance, he does not fail to pronounce his name; it only appears of consequence to him to reproduce the word which he may have just heard. If his instructor would wish to address himself to his mind, he employs for the purpose gestures or expressions of the countenance. It is by signs only that the youth expresses himself with ease and quickness, and it is only by the employment of these signs that a judgment can be formed of his intelligence, and of the quickness of his conceptions.

In this point of view, Honoré presents a phenomenon highly interesting. Having acquired a new medium for the expression of his ideas, it might be supposed that he would have neglected the method, so greatly inferior to speech, of which, until then, he had availed himself. But, up to the present time, the contrary has happened; his natural language, namely, that of signs, instead of being lost and gradually replaced by speech, has rapidly improved, and acquired a perfection and quickness much superior to that which it displayed before he had recovered his hearing.

However, in his connexions with children of his own age, Honoré begins to employ simple words, and particularly substantives, to make known his principal desires. Perhaps the time may arrive when he will make a more frequent and complete use of speech; but in this respect it is possible that he may always remain far below other men. For we have numerous examples of children who may be called dumb, only because there requires in them a certain effort of the ear to comprehend words, and rather a difficult exertion of the larynx to speak. Finding, by the employment of signs, an easy medium of communication, they neglect to exercise the ear and the organs of speech, and thus remain classed among the deaf and dumb, when in reality they are neither dumb nor deaf.

But to return from this digression.—Honoré Trezel, who had been completely deaf, even to such a degree that a year ago he did not hear the loudest detonations, now listens attentively to all noises, knows when they come from afar off, distinguishes their character, avoids carriages and horses, and goes to open a door when any person knocks. He knows how to appreciate the musical rhythm, and takes pleasure in listening to singing and musical instruments; he tries even to imitate the modulated voice, without, however, having been yet able to succeed. He knows how to appreciate and repeat all the articulations of our language; he comprehends, he analyses, repeats from memory a certain number of phrases within his capacity, and he replies to them. He executes what his instructor by words directs him, but with other persons he is not yet able to do this; for the same reason that we comprehend a foreigner when we are accustomed to his pronunciation, and for the same reason that we are entirely incapable of comprehending him when he speaks to us for the first time.

Here are assuredly results sufficiently gratifying. When we take into consideration all that this child has learned before arriving at his present condition, as well as all the ideas and new combinations of them which have been indebted for their operations in his mind to the instinctive associations which have been established between his ear and his intelligence,—between these, again, and the organs of voice,—between his ear and his larynx, we have no difficulty in flattering ourselves with the hope that his moral condition and his physical state will continue to improve.

But let us not anticipate the future; let us rather wait the result of experience, which in this, as in all new questions, can alone enlighten us.

Your commissioners think that the efforts of M. Deleau to impart the blessings of social life to beings who, in a great degree, had been separated from it by nature, are deserving of the eulogium of the Academy; that the results at which he has arrived in the young Trezel, are very important, and worthy of the most lively interest. They propose to you to engage M. Deleau to continue the education that he has so happily begun; to multiply, as much as possible, observations of the same kind; and thus to lay the foundation of a species of instruction or education, which ought to be esteemed among the number that ameliorate the condition of the human race.

(Signed) DUMERIL,
GEOFFRY ST. HILAIRE,
MAGENDIE, *Reporter.*

---

ART. II.—*Case of a Wounded Nerve, followed by severe Consequences, and cured by removing the Wounded Portion of the Nerve.* By GEORGE BELL, Esq. F.R.S.E.; Fellow of the Royal Colleges of Surgeons, Edinburgh and London; Surgeon Extraordinary to the King, and Surgeon in Ordinary to His Majesty's Household for Scotland. (From the same.)

THE affections and injuries of the Nerves, including contusions, wounds, and the different varieties of tic douloureux, which fall, almost exclusively, under the care of the surgeon, have always excited much interest and anxiety, both in the patient and practitioner, not only in consequence of the extreme pain usually accompanying them, and the uncertainty of the influence of the remedies employed, but of the long continuance of the symptoms, the ill health, and sometimes the fatal consequences resulting from them.

As the older anatomists were unacquainted with the minute anatomy of the nervous system, we need not be surprised that the contemporary surgeons should be ignorant of its pathology; and, therefore, that their treatment of its chirurgical diseases should be conducted upon almost empirical principles. Thus we find a number of detached cases, dispersed in various books of general surgery, as well as in the Ephemerides and other periodical publications on the continent, and in this country, treated as diseases or injuries of the nerves, where, in fact, the diseases ought, in many instances, to have been sought for in the veins, the aponeurotic expansion, or in an erysipelatous affection of some other tissue. But the indefatigable exertions and accurate investigations of Wrisberg, Monro, Reil, and other great anatomists of the last century, followed more lately by the researches of Bichat, Sir Everard Home, Scarpa, Mr. Charles Bell, and others, have cleared away many of the difficulties connected with the anatomy, and rendered the pathology of the tissues so intelligible, that we may not despair of seeing the treatment of chirurgical diseases, and affections of the nerves, reduced to as great a degree of simplicity, and to as fixed principles, as many others coming under the care of the surgeon. More especially, I cannot help anticipating great improvement, not only in the chirurgical but in the medical treatment of nervous affections, from the labours of Mr. Charles Bell, when his Exposition of the Nervous System comes to be generally read and understood.

In the mean while, I am persuaded that the faithful narrative of unequivocal cases, may, in this as in other subjects, induce practitioners to bring forward those of the same kind they have met with, and thus form a body of detached information, which may be classed when our knowledge is more matured. With this view I am induced to offer the history of a case, where, I have every reason to believe, a nerve was punctured in the operation of bleeding, where the symptoms soon became extremely urgent and interesting, and where a cure was effected by a complete division and removal of the parts surrounding the wound.

On the 7th July, 1802, I was desired by Dr. James Hamilton, professor of midwifery in this University, to visit with him a young woman, who had been bled ten days before in the median cephalic vein of the right arm.

When I saw her, the tendon of the biceps was much contracted, and the forearm bent to an acute angle with the arm. The fingers also were firmly clenched, and she could neither allow them or the arm to be extended, without suffering the most excruciating pain. On examining the arm, no swelling of it was perceptible, and the wound of the vein was completely healed; but, when this spot was even gently pressed, she complained of great uneasiness. The pain was not confined, however, to the neighbourhood of the wound, but extended down the forearm to the very tips of the fingers, and stretched upwards along the inside of the arm to the axilla, thence to the clavicle, pectoral muscle, and even the short ribs.

The pain was, to a certain extent, constant, but occasionally considerable exacerbations were experienced; and at these times she was seized with startings, tremors, and subsultus tendinum, and other nervous symptoms. The pulse was 110; she was hot and restless, her tongue dry and parched; she had a general uneasiness over her whole body; in short, all the symptoms of fever. As she was of an irritable habit, and easily affected by slight impressions, and every internal medicine and external application that Dr. Hamilton could think of had been used, without producing any sensible good effect, before I saw her, there was much reason to apprehend that tetanus or locked jaw would soon supervene, unless relief were obtained from the division of the nerve which we had no doubt had been wounded.

As I was obliged to perform the operation by candle-light, when it would be extremely difficult, if not impossible, to dissect from the neighbouring parts so minute a nerve as that which probably was wounded, I determined to remove a considerable portion of the vein which had been opened, so that I might be certain of including the injured nerve within the two incisions.

I was induced to adopt this plan from another consideration, as it occurred to me that there was little chance of my being able to divide the nerve precisely at the place where it had been punctured; and, if it was only divided in one place, either above or below the puncture, or even both above and below, the numerous anastomoses which connected it with other cutaneous nerves might prevent the success of the operation.

With this view, and with the concurrence of my friends, Dr. Barclay, who attended in consultation, and Dr. Hamilton, an incision, about three inches in length, was made through the skin along the course of the vein, commencing an inch and a half above, and terminating at the same distance below, the wound which had been made by the lancet. The vein being laid bare, and separated from the tendinous aponeurosis, two ligatures were thrown round it, at an inch and a half from each other, and equi-distant from the wound of the vein. The ligatures were then tightened, and the intermediate portion of vein divided as close to them as possible, and removed.

When the ligature was tightened on the upper part of the vein, she complained of considerable uneasiness, much greater than that which she felt on tightening the ligature on the lower part.

On dividing the vein, the cut ends retracted to a considerable distance from each other, and the pain which she felt previous to the operation immediately disappeared. She moved her fingers with the greatest freedom, extended and bent her arm, and performed all the motions of the wrist and elbow joints with perfect facility. The pain of the side, of the axilla, and all those symptoms which rendered the operation necessary, seemed to be removed; and she now complained of no farther uneasiness than that which a wound of equal extent in any other part of the body would have occasioned.

The sides of the wound were drawn together, and retained in contact by slips of adhesive plaster; a pledget, compress, and bandage, completed the dressings. She was placed in bed with her arm extended on a pillow, in such a manner that the flexors of the forearm were completely relaxed.

On the following morning (8th) I found she had slept well, by the aid of an opiate: the sleep had not been disturbed by any startings, tremors, or other nervous sensations, with which she had been affected previous to the operation. She lay as easy and was as comfortable as could be expected, considering the constrained posture in which she was placed. During the night, however, her fingers were again contracted, probably from the superior power of, or irritation applied to, the flexor muscles, or possibly from the irritation excited by the ligatures of the vein; but, although she could not extend them at pleasure with ease, she could permit them to be moved in every direction, without increase of pain.

On the 9th, she complained of slight uneasiness in the fingers and forearm; but these sensations seem to have been occasioned by her having passed a restless night, and having been obstinately costive. A dose of compound powder of jalap was therefore given, which operated copiously in the course of the day, and an emollient poultice was applied to the wound.

10th.—She slept well during the night; has no symptoms of fever; the pain of the arm relieved, and the wound has an healing appearance.

12th.—Remains easy and free from fever. Ligatures were removed this forenoon, and the pain which the upper one seemed to have occasioned is gone. The wound is now dressed with adhesive plasters.

From this time she continued quite well as to her general health, complaining occasionally of a sense of stiffness or numbness in her arm, though not to a greater extent than was reasonably to be referred to the wound made in the operation. The wound was healed on the 1st August.

In this case, I have no doubt that at least a permanent contraction of the elbow-joint, if not an attack of tetanus, was prevented by the operation having been performed so soon after the infliction of the injury; and I would be disposed to hold it out as a case affording encouragement not to delay removing a portion of a partially wounded nerve, producing symptoms similar to those above detailed, where the nerve is accessible to the knife.

But, although an early operation is in all cases advisable, yet, if it has been neglected or unavoidably postponed, the experiment ought to be made even at a very protracted period from the accident, and when symptoms which, when idiopathic, are generally considered incurable, have supervened. The propriety of this is exemplified by the following case:—

A young woman, sixteen years of age, when engaged in her domestic duties, cut herself with a knife, about three fingers' breadth above the wrist, wounding both the artery and nerve. The arterial blood was soon stemmed, the wound healed; and there remained only a little pustule, resembling a bilberry. Some few months afterwards she became affected with fainting fits, and applied to Volchamer for advice. He inquired minutely into the history of her complaints, and suspected an injury on the head; but, on being shown her arm, to the injury on which, and the loss of blood, she attributed her complaints, he (taking the pustule for an incipient aneurism) directed a surgeon to apply caustic, which was done so effectually that a large eschar separated. The wound was kept open as an issue for six months, and the young woman had no return of fainting fits.

Volchamer observes that, unless you cut out, or burn out, a wounded nerve, convulsions are very likely to take place.

A case of the same description, but with a different result, has been kindly pointed out to me by Dr. Milligan, who was a witness to the progress of the symptoms while the lady was in this country, and has repeatedly heard of her since her return to Ireland, where she still resides.

This lady, at the age of twenty-two, was bled on the 7th June, 1819, in the median basilic vein of the left arm, for a stitch in the left side. She immediately complained of pain in the wound; went to bed three hours afterwards, and soon experienced extreme uneasiness in the left shoulder. At three in the morning, she was attacked suddenly with spasms of the extensors of the hands and nerves; great anxiety, restlessness; pain at the scrobiculus cordis, increasing for several hours, when the muscles of the neck and back became affected, and the body bent backwards, as in opisthotonos; the muscles of the upper and lower extremities, and the pectorals, acting in the most violent manner. These spasms continued for six minutes, and then went off. She took large doses of laudanum from the commencement; 580 drops being given within the first twenty-four hours without relief, the spasms returning frequently during the day and night. These continued for a very long period to return incessantly; so that she took 44,000 drops of laudanum in the space of two months; and she is to this day annoyed by frequent renewals of the disease, whenever she is agitated by any vexatious or unforeseen occurrence.

In this case, if a portion of the vein had been removed within a moderate period after the wound was inflicted, she might have been relieved at least of a part of her suffering. And even at a more distant period, as in the case related of Volchamer's, I would not despair, but would give the patient a chance for being cured, unless some important contra-indication should present itself. This leads me to detail briefly the outlines of another case of wounded nerve, which occurred to me early in practice, and where the patient recovered from a long continued and highly excruciating disease.

In the month of June, 1805, a lady, about twenty-six years of age, the wife of a surgeon in Lincolnshire, applied to me, when suffering severely under symptoms of violent nervous irritation, apparently connected with a wound which she had received two years before, when cutting a loaf of bread; the knife having slipped and divided the artery, and probably injured the nerve of the thumb, on the radial side of it, half way between the first and second joints. The pain was excruciating at the time, but the wound healed kindly; yet the pain continued, and was accompanied by startings, twitchings of the flexors, bending of the thumb, and great general irritability. Various opiates, without large doses of which she had never slept since the reception of the injury, and every expedient that could be thought of, were employed by her husband and the medical gentlemen who attended her; and on two different occasions, at a considerable intervening interval, an incision was made through the soft parts to the bone in the neighbourhood of the wound, but with slight and only temporary relief. When she came to Edinburgh, her general health had suffered much, from the long continuance of the painful spasmodic symptoms; and now the pain was sometimes so exquisite, and her mind had become so irritable, that I dreaded mental derangement would ensue. Before removing the thumb at the second joint, I requested Dr. Monro (secundus) and Mr. Russell to see her. Mercury was proposed, and tried; but the symptoms increased, and we were glad to lay it aside. When the mercury seemed to have left the system, the thumb was removed; the twitchings and painful contractions were soon relieved; her mind became less irritable, and she returned home in five or six weeks, restored to perfect health.

I consider the cases which I have just briefly detailed, as well marked examples of nervous or tetanic affections produced by a partial division or injury of a nerve. I am aware, indeed, that the wound or prick of the nerve was not demonstrated by dissection, and made visible to the eye; and it must be obvious to every practical surgeon, that such demonstration would, in almost every instance, be impracticable, both from the minuteness of the object, the perpetual flow of blood, and the tedious dissection in the living body: so difficult, indeed, would such demonstration prove, that I doubt whether it has ever been accomplished in more than one case yet on record. But these cases, and some others which have been recorded, afford sufficient data for distinguishing wounds of the nerves from those cases of inflamed veins, so well described by Mr. John Hunter, and from the inflammation of the surrounding cellular membrane, described by Dr. Duncan; and for establishing, as a safe rule of practice, the division or the destruction of the affected portion of the nerve, if within the reach of the knife or cautery, as soon as the nature of the injury is ascertained,—if possible, within a few days: yet not to be deterred by the lapse of any length of time from the reception of the injury, provided no constitutional disease shall contra-indicate an operation.

I will only further add, that the probability of success in such cases is infinitely greater than in those where the nerve is divided in tic douloureux; for we do not yet know whether the cause of this last disease resides in the extremity of the apparently affected nerve, or at some spot between the extremity and its origin in the sensorium. In the former case, we are quite certain of removing the affected portion of nerve; but, in tic douloureux, we can never be sure of being able to reach the part that is diseased, until the success of the operation proves that we have done so.

---

ART. III.—*Of the Expectoration in Phthisis.* (From the Thesis of M. ANDRAL.)*

WE think, with Aretæus, that it is by the consideration of their *physical* properties that the sputa peculiar to the tubercular degeneration of the lungs, can be best recognised.

At the commencement of phthisis, when some lesion of the pulmonary organs, more important than simple catarrh, is indicated by cough with frequent hæmoptysis, emaciation, and irregular febrile attacks, the sputa are yet without character.

In most individuals the cough is dry, while in others it is accompanied by a purely catarrhal expectoration, which, although remaining for a length of time, still preserves the character of that in acute catarrh. This circumstance is not to be overlooked, and should cause the physician to suspect the existence of tubercles. Nevertheless, after this dubious catarrh has remained for a long time, if the sputa are examined daily, small yellowish-white grains are observed in the expectorated matter, which have a tolerable consistence; and vary from the size of a pin's-head to that of a pea. They remain separate, and fall to the bottom of the vessel; when broken, they exhale a very fœtid odour, which has been regarded by Baglivi as pathognomic of pulmonary consumption.

We must not confound these granular bodies with those secreted by the pharyngeal glands, which are exceedingly viscid and tenacious, presenting a strong contrast to the friable tubercular debris.†

At the same period of the disease, the sputa are sometimes composed of a transparent colourless liquid, in which are suspended long and delicate striæ; in some instances floating on the surface of an opaque

---

* From Dr. STOKES' *Introduction to the Use of the Stethoscope.*

† A better method of distinguishing these substances is by heating them on paper. The secretion of the tonsils and neighbouring glands is sebaceous, and therefore greases the paper. This is not the case with the tubercular matter.

mucus, from which they may be distinguished by their dull white colour.

If death takes place during the first period, the lungs are found studded with small tubercles, some of which are hard, while others are beginning to soften towards their centre. It is rare, at this period, to meet with tubercles completely softened, or excavations of any size.

We sometimes meet with patients who, having long laboured under a dry cough, with all the other symptoms indicative of the existence of crude tubercles in the lung, suddenly expectorate a large quantity of puriform sputa, coming from a tuberculous excavation, which had opened into one of the bronchial tubes. This circumstance may prove fatal; but Bonet and Laennec relate two cases where it took place, and yet the patients recovered their health.

According as the grains and delicate filaments, which we have described, become more abundant, they unite into different-sized masses, which remain suspended in the midst of a clouded serosity; these may be then called *flocculent sputa.* In other instances they appear round and thickened, remaining perfectly separate from one another; these we have called *nummular sputa.* The appearance of these sputa is regarded by Quarin as one of the most fatal symptoms. He states that he has never seen a case terminate favourably in which they were present. When examined with the naked eye, they are seen to be formed by a multitude of little points, capable of still farther subdivision. These whitish molecules are united by a semi-transparent greyish mucus, which sometimes, however, is yellow or greenish, and completely opaque; so that the expectoration has a variegated appearance. At other times the sputa are composed of long delicate striæ, sometimes twisted on themselves; and united by mucus, from which they are distinguished by their colour. But at this period the expectoration presents innumerable shades of difference, which depend, first, on the manner of communication of the bronchial tubes with the tubercular excavation; second, on the number, length, size, and mode of division of the bronchial tubes, through which the matter must pass before coming to the trachea; third, on the quantity and nature of the mucus with which it is mixed; and, fourth, on the length of time it has remained in the bronchial tubes before its expulsion.

In whatever manner these varietes are formed, we obtain a correct idea of them from the name of *compound sputa,* first given by M. Lerminier. When a patient dies who presented the above expectoration, we are sure to find the tubercles in a complete state of softening, with deep excavations already formed in the pulmonary tissue.

During the latter periods of the disease, there is secreted from the sides of the tuberculous excavations a liquid of a dirty ash-grey, sometimes reddish colour, which last tinge appears to arise from the mixture of a certain quantity of blood. This liquid, which has a great analogy to the sanious pus of old and ill-conditioned ulcers, is frequently mixed with small grains of decomposed tuberculous matter.

When the excavations are found containing the above fluid, it is generally the case that its existence was revealed by the characters of the expectoration; in which latter, this liquid occurs at first in small quantity, but, gradually increasing, at length almost entirely constitutes it. It is then nearly a homogeneous pus, sometimes fœtid, sometimes inodorous, and containing grains of softened tuberculous matter scattered through it. These latter, however, do not always occur; and we have seen cases where, although found in the excavations, they did not appear in the sputa.

In the latter periods of the disease, when the sputa do not take on the puriform aspect, but still continue divided, it frequently happens that, twenty-four or forty-eight hours before death, the character of the expectoration is altogether changed, the serosity disappears, and the sputa form a thick greyish mass, strongly adherent to the bottom of the vessel.

In other cases, expectoration is altogether suppressed a short time before death; the symptoms are then aggravated, and the strength rapidly diminishes. This sudden suppression may be justly looked upon as one of the most fatal symptoms. As in pneumonia, it may arise from two causes: first, from the inability of the patient to expectorate, the sputa collect in the larynx and trachea, and he sinks in a state of asphyxia. In the second case, the expectoration is suddenly suppressed, without any tracheal rale being heard, while at the same time the mucous rale, which indicates the existence of a cavity filled with liquid under the point where it occurs, suddenly ceases to be heard in this situation, although a short time before it had been completely evident. We must then admit that the liquid filling the cavity was rapidly absorbed.* In some cases, where the patients ceased to expectorate immediately before death, we have found vast excavations entirely empty.

The attention of physicians has been for a long time directed to the odour of phthisical sputa, which in most patients have a faint and nauseous smell. In these individuals, the disease may go through its different stages, and death supervene, without the odour of the sputa becoming more disagreeable. In other cases, the expectoration, although long inodorous, will, a few days before death, acquire an insupportable fœtor, which is also perceived in the matter of the cavities.

What is the cause of this change of odour? The fœtor of pus, which often arises from its contact with air, may in some cases depend on other circumstances; such as its own qualities, and the time it has remained in the cavity. The same should be true of the liquid contained in tuberculous excavations of the lung.

But observation has shown that, from whatever cause the fœtor may arise, it is one of the most fatal symptoms, when occurring in a patient whose expectoration was previously inodorous.

The fœtor of expectoration, at the commencement of the disease, is a far less unfavourable symptom; as patients thus affected have lived for a length of time.

The taste of the sputa, as perceived by the patient, has attracted as much attention as their odour. Most authors have advanced, that those patients whose sputa are insipid sink less rapidly into a state of maras-

---

* May not the phenomenon be explained, by supposing that the softened tuberculous matter has passed into and filled the smaller bronchial tubes, from whence, owing to the great debility of the patient, it could not be expectorated?

mus. But our experience does not confirm the truth of this position. We have seen many phthisical patients who complained of the insupportable taste of their expectoration, and who nevertheless sunk but slowly. Others, on the contrary, died rapidly, although their sputa were nearly insipid. We have met with few patients whose expectoration had the mild and saccharine taste described by Hippocrates as one of the symptoms of pulmonary consumption. Sputa possessed of a saline taste, have also been noticed by Hippocrates as one of the precursory signs of phthisis. Morton insists much on this symptom; but we see many patients labouring under a simple catarrh, whose sputa have a saline taste, and yet these individuals may never become phthisical; and, on the other hand, there are many consumptive patients whose sputa never have a well-marked saline taste.

It must be left to future experience to decide whether the expectoration of phthisical patients can ever be so acrid as to erode the parts with which it may come in contact.

Hitherto we have described the expectoration in phthisis as it occurs in the majority of cases. But we have seen several instances where this disease went through all its stages, where, even after death, large excavations were found in the lung, and in which, notwithstanding, the expectoration furnished no diagnostic sign whatever. In support of this singular fact, I shall quote the following cases:—

1. A young woman, labouring under violent cough and dyspnœa, remained for three weeks in the hospital, with evident pectoriloquism in the antero-superior part of the right side of the chest. Her expectoration, however, was all through purely catarrhal, and composed of a colourless, transparent, and thready mucus, adherent to the edges of the vessel by long striæ, some of which were opaque. After death, large excavations were found in the right lung, half filled with an ash-coloured liquid, through which there floated small grains of a dull white colour. Both lungs were studded with tubercles, most of which were still crude.

2. A young man, presenting every symptom of phthisis in its last degree, with pectoriloquism at the sub-spinous fossa, had habitually but a trifling expectoration, similar to that of acute catarrh arrived at its last period. It was composed of a greenish yellow, thready, homogeneous mucus, mixed with air and saliva. During the six weeks that he remained in the hospital, his expectoration was mixed with striæ, of a dirty reddish colour, which appeared to be derived from pulmonary excavations. Upon dissection, we found the right lung entirely studded with miliary tubercles, of which some were also seen in the left pulmonary organ. At the superior part of the right was a cavity capable of containing a large walnut, and completely empty.

3. Upon opening the body of a woman who had sunk under a chronic diarrhœa, we found at the superior part of both lungs several small cavities, which communicated with one another, and were filled with softened tuberculous matter. Into one of these a large bronchial tube opened. But it is extraordinary that, during this woman's stay of three weeks in the hospital, she never expectorated at all, nor complained of any morbid symptom in the side of her chest.

A young man sunk under peritonitis, which supervened on a chronic enteritis. He stated that he never coughed, and the expectoration was wanting. After death, an empty cavity, capable of containing an apple, and lined by a thin concrete purulent matter, was found at the top of the right lung. Several smaller excavations, communicating with one another, and equally empty, were found on the same side. The rest of the lung contained many crude tubercles.

In this case, did the excavations, once emptied of their tuberculous matter, cease to produce any new secretion? Was the secretion in the lung suspended by the abdominal affection?

In some instances, we have found large excavations, filled with a grey or reddish liquid, which was not observed in the sputa, although they indicated clearly the existence of phthisis.

When pneumonia supervenes on phthisis, the sputa change their character altogether, and only show acute inflammation of the lung; except in some cases, where the two expectorations are blended.

In order that we shall not be led into error by the characters in phthisical sputa, it is necessary to pay attention to the particular hour at which they are examined. Many patients have a characteristic expectoration during the night and morning only, and for the rest of the day they do not expectorate; or, if they do, their sputa are purely catarrhal. Others only expectorate at the close of the hectic exacerbations, while during the paroxysm their cough remains dry.

Tuberculous excavations are thus analogous to many external ulcers, whose surfaces become dry during the access of an intermittent, and again moist at the close of the paroxysm.

It sometimes happens that, at intervals, the sputa cease to be characteristic, without our being able to assign any cause for this change. This intermittance of expectoration, we have frequently seen to alternate with diarrhœa; when the stools became very frequent, the sputa were either less abundant, or ceased altogether. We have also seen a case of a phthisical patient, who had caries of one of the left ribs, in whom the expectoration diminished whenever the suppuration increased, and vice versâ.

### *Expectoration in Emphysema of the Lung.*

Emphysema of the lung is generally accompanied by the symptoms of catarrh, and the expectoration is therefore very variable in its character; the cough is sometimes dry, sometimes followed by the expulsion of a greyish, transparent, and more or less viscid fluid. At other times the sputa are thick and opaque.

### *Expectoration in Œdema of the Lung.*

In this case the expectoration, according to M. Laennec, is aqueous and transparent, like that of acute catarrh. But, as œdema of the lung is rarely a primitive disease, and may occur in many different diseases where the bronchial membrane is already affected,—such as chronic catarrh, pneumonia passing into resolution, diseases of the heart, fevers, &c.—it seems probable that the expectoration in this disease may appear under many different aspects; and such, indeed, has been the result of our experience.

